# The effect of selenium supplementation on cystine crystal volume in patients with cystinuria

**DOI:** 10.1051/bmdcn/2018080426

**Published:** 2018-11-26

**Authors:** Mehrdad Mohammadi, Amin Shohani, Hatef Khorami, Kia Nouri Mahdavi, Mohammad hossein IzadPanahi, Farshid Alizadeh, Mohammad Azizi

**Affiliations:** 1 Isfahan Kidney Transplantation Research Center, Department of Urology, Alzahra Research Centers, Isfahan University of Medical Science Isfahan Iran

**Keywords:** Cystine crystal, Cystinuria, Selenium

## Abstract

Background: Cystinuria as an autosomal recessive sickness is a relatively rare disease. Formation of cystine stones indicates cystinuria. Few studies are considered the cysteine crystal volume in management of cystinuria. Selenium may inhibit organization of crystal stone, growth and stone aggregation. Since the role of selenium on inhibition of cystine crystal formation is not known, the aim of this study was to evaluate the effect of selenium supplementation on cystine crystals volume in patients with cystinuria.

Materials and methods: This double-blind clinical trial study was conducted on 48 patients in Al-Zahra hospital, Isfahan, Iran from 2015 to 2017. These patients received selenium (200 mg/ daily) for 6 weeks. The urine crystal volume was evaluated before and after treatment. Data were entered SPSS and analyzed by Paired sample *T* test, Spearman and Pearson coefficient correlation. *P*- value < 0.05 was considered significant.

Results: In current study, mean cystine crystal volume before and after treatment was 6787.4 ± 11902.6 and 3110.9 ± 7225.4, respectively. Significant difference was observed before and after treatment in terms of cystine crystal volume *(p* < 0.001). No relation was observed between the mean cystine crystal volume with sex, age and type of medical procedures *(p* > 0.05).

Conclusion: In this study, selenium treatment affected cystine crystal volume. It seems that selenium had the potential value to alleviate the volume of cystine crystal. Therefore, since reducing of cystine crystal volume decreases crystal formation, selenium may be effective to cure patients with cystinuria. However, age, sex and type of medical procedures did not affect cysteine crystal volume.

## 1. Introduction

Cystinuria as an autosomal recessive sickness is a relatively rare illness. Its incidence is 1:7000 [1], but its distribution varies in population in terms of genetics and ethnic. Prevalence range is 1:2,500 in the Libyan Jewish population, 1:100,000 in Scandinavian population and 1:15,000 the US [2]. In this disease, reabsorption of cysteine and basic amino acid from renal proximal tubule and small intestine is heredity defected. Formation of cysteine through disulfide bond stones indicates cystinuria [1]. The elevated cysteine excretion through urine leads to cystine crystallization and stone formation [1]. Cystine stones are observed in 1% to 2% of patients with nephrolithiasis and 6% to 8% of stones in children [3–5]. Moreover, patients with cystine stones have more chance of chronic kidney disease than patients with calcium oxalate stones [6, 7]. The most age range of stone formation is observed between 30-40 years old, although stone formation can happen at any age [3]. Diverse factors can affect solubility of cysteine including cystine concentration and ionic strength [8].

Since cystine stones mostly are large and resistant to disruption through shock wave lithotripsy, other methods including surgery may be a usual treatment [9]. Because growth of stones and formation of new stones are rapid, they need several surgical treatments [9–11]. Other common treatments include increasing urine volume, restricting sodium diet and alkalising urine. Furthermore, the most ordinary treatment in the management of cystinuria is D-penicillamine, however, it is associated with side effects such as proteinuria, particularly rash and fever. Moreover, Pruritus and arthropathy are other side effects of cystinuria [12]. Determination of crystal cysteine volume can predict activity of crystal formation in cystinuric patients. Therefore, decreased volume of cystine crystals can be another useful tool in the management of cystinuric patients [13]. Recently, some studies have shown that selenium is able to suppress renal stone formation. It seems that selenium inhibitory activity is due to binding of this compound onto the crystal surface. Selenium can inhibit organization of new crystals, growth and aggregation [13]. Moreover, selenium as an antioxidant can also form a bond with cystein nominated selenocysteine (one of the features of cystein is metal binding). Seleno-cysteine can decrease the disulfide bond through exchanging disulfide-bond by selenylsulfide bond [14]. Object of Cystinuria treatment is to reduce the urinary concentration of cystin under to its solubility limit (200 to 300 mg/*l*).

Despite the morbidity due to cystinuria, its treatment has not been changed during these years [7, 15, 6]. At present, the evaluation of patients with cystinuria is based on the total amount of urine cysteine within 24 h. Moreover, none study is considered the cysteine crystal volume in management of cystinuria in our country and very few studies have evaluated the role of selenium for suppressing cystine crystal formation. Furthermore, because of the importance of cystine crystal formation in cystinuria patients, the aim of this study was to evaluate the effect of selenium supplementation on cystine crystal volume in cystinuria patients.

## 2. Methods

### 2.1 Study design and population

This double-blind clinical trial study was conducted on 48 patients in Al-Zahra hospital, Isfahan, Iran during 2015 to 2017. After taking consent from patients, this study was approved by ethnic committee of Isfahan University of Medical Sciences (number: 295163). These patients received selenium (200 mg/ daily) for 6 weeks. Demographic data, family history and previous treatment were obtained from all patients and recorded in checklist.

### 2.2 Inclusion and exclusion criteria

Inclusion criteria were diagnosis of cysteine stone. Moreover, exclusion criteria were using of other medications or mineral except selenium.

### 2.3 Cystine crystal volume determination

Before and after treatment, urine was centrifuged (2000 g for 5 min) and sedimentation evaluated for studies. Then we investigated neo-bar lam by 40-fold magnification. Lam (neobar lam) was used for calculation of the volume and number of crystals. Due to the scalability of the lam, the distance between the two opposite angles of the crystal was measured and placed in the following formula.

The cystine crystal volume was evaluated before and after treatment by following formula.

$$\mathrm{Volume}\;\mathrm{of}\;\mathrm{cystine}\;\mathrm{crystal}\;=\;0.625\;\times \;N\;\times \;{\left(L\right)}^{2}\times \;T\;(17)$$

N = The number of crystal

L: The mean length of the two opposite angles in the crystals

T: Average thickness of the crystals (constant)

To calculate T, L and number of crystals, we calculated the crystal size in term of mm^3^, L and T in terms of μ. Urine was placed in neobar lam and crystals observed by microscope.

Volume of crystals was obtained in terms of μ^3^/ mm^3^. Since average thickness of the crystals was not evaluated by this method, T was considered constant.

Moreover, cystine crystal volume below cut off 3000 indicated the low chance of stone recurrence.

### 2.4 Statistical analysis

Data were entered SPSS version 21 and analyzed by paired *T* test, spearman and Pearson coefficient correlation. P-value < 0.05 was considered statistically significant.

## 3 Results

### 3.1 Demographic characteristics of the study population

In current study, among 48 patients with cystinuria, 23 patients are male and 25 female. The mean age of patients was 39.2 ± 12.3 years old. These patients underwent surgery several times in a lifetime.

### 3.2 Mean number of surgical procedure in patients with cystinuria

Mean number of surgical procedure in these patients is shown in Table 1. As shown in Table 1, the most medical procedure which was performed in these patients was Transureteral lithotripsy (4.04 ± 2.14 times) and the least used surgery was due to Open laparoscopy (Open. S) (0.47 ± 0.87 times).

**Table 1:** Mean surgical number in patients with cystinuria.

Types of surgical procedures	Mean
Trans ureteral lithotripsy (TUL)	4.04 ± 2.14
Percutaneous nephrolithotomy (PCNL)	2.27 ± 1.18
Extracorporeal Shock wave lithotripsy (ESWL)	3.27 ± 2.52
Open laparoscopy (Open. S)	0.47 ± 0.87

### 3.3 Relation between the type of medical procedures with mean crystal volume

Relation between type of medical procedures with mean crystal volume before treatment showed that no relation was observed between mean crystal volume with Trans ureteral lithotripsy, Percutaneous nephrolithotomy, Extracorporeal Shock wave lithotripsy and Open laparoscopy (Open. S) (Table 2) *(p* > 0.05).

**Table 2:** Relation between the type of surgical procedures with mean crystal volume.

Types of surgical procedures	Mean crystal volume
	*p*-value
Trans ureteral lithotripsy (TUL)	0.33
Percutaneous nephrolithotomy (PCNL)	0.40
Extracorporeal Shock wave lithotripsy (ESWL)	0.61
Open laparoscopy (Open. S)	0.96

### 3.4 Mean cystine crystal volume

**Fig. 1:**
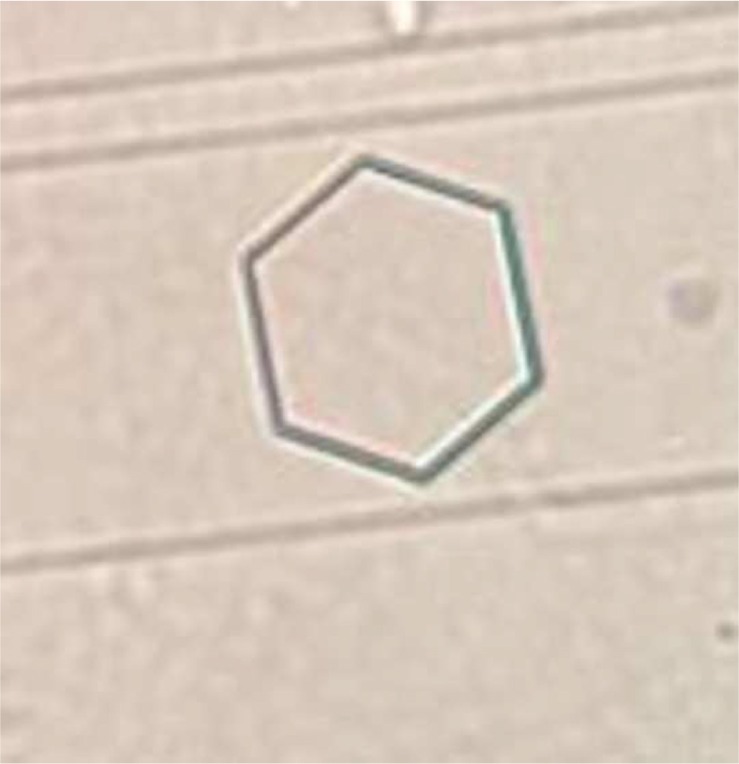
cystine crystal in urine. (40×)

**Fig. 2:**
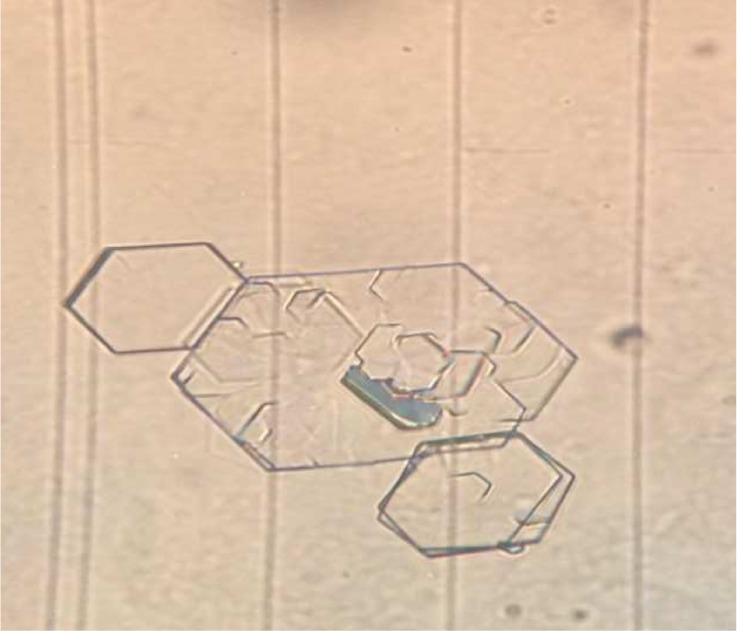
adhesion of crystals to each other.

The mean cystine crystal volume in urine samples before and after treatment is shown in Table 3. As shown in Table 3, significant difference was observed between mean cystine crystal volume before and after treatment *(p* < 0.01). Fig. 1 shows cystine crystal in urine. As shown in Fig. 1, 6 sided crystals were cyctine stone crystals. Fig. 2 shows the adhesion of the crystals to each other. When crystal volume is greater, the chance of sticking to each other is greater.

**Table 3:** The mean cystine crystal volume before and after treatment.

Mean cystine crystal volume (before treatment)	Mean cystine crystal volume (After treatment)	*p*-value
6787.4 ± 11902.6 μ^3^/ mm^3^	3110.9 ± 7225.4 μ^3^/ mm^3^	0.000

### 3.5 Relation between the mean cystine crystal volume with sex and age

Moreover, relation between the mean cystine crystal volume with sex and age is shown in Table 4. As shown in table 4, no relation was observed between the mean cystine crystal volume with sex and age *(P* > 0.05).

**Table 4:** Relation between the mean cystine crystal volume with sex and age.

Parameters	Mean cystine crystal volume
	*p*-value
age	0.24
sex	0.16

## 4. Discussion

Selenium acts as an antioxidant with anti-inflammatory feature in selenoprotein form. It as a glutathione peroxidase (GPx) enzyme component decreases reactive oxygen species (ROS) such as H2O2, and ROOH in the body *via* enzymatic reactions [18]. It has a preservative role in diverse diseases including cancers and urological disorders. The role of selenium in cystine crystal formation is poorly documented [19].

Current study was performed for the first time in Iran. This study considered crystal volume as a good appliance in the assessment of patients with cystinuria and showed that selenium supplementation significantly decreased cystine crystal volume. Chrzan *et al.,* in 2015 reported that changing of atomic structure of cystine *via* substituting of sulfur by selenium causes distortion cystine crystal, increases solubility and decreases precipitation. This model can serve a plan for future drug progression [20]. Singh *et al.,* reported that the function of selenium is prevention of stone formation [21]. Salky *et al.,* in 2003 reported selenium is able to inhibit calculogenesis. Nahas *et al.,* observed that the use of antioxidants like selenium and vitamin E protected the renal tissue from inefficacious effects caused from the free radical activates [14]. Ludwing *et al.,* in another study reported that selenium supports kidney cells versus shock-wave-induced injury. Since selenium constitutes glutathione peroxidase fragment, it may done *via* reactive oxygen species (ROS) reduction [22]. Liu *et al.,* confirmed previous studies and reported that selenium supplementation may increase antioxidant capability, reduce oxidative damage and decrease calcium oxalate renal calculi in dogs [23, 24]. Grases *et al.,* demonstrated that increased level of urinary free radicals is associated with cell injury, resulting in an appropriate surroundings of crystal formation [25]. It is interesting that oxygen free radical has a main role in renal tubular. It may have an effect in renal calcium oxalate calculi formation, while antioxidant *via* prevention of calcium oxalate crystal formation exerts its effect. It shows the application of selenium may be lucrative.

Experimental and animal model demonstrated that oxidative stress leads to renal tubular lesion. It is a leader factor for calcium oxalate calculi organization [26]. As the important inducement of calcium oxalate calculi organization sustained hyperoxaluria causes renal tubular epithelial cell injury and phosphatidylserine leads to crystal adhesion [23]. Other studies also reported that Vitamin E and selenium decreased the occurrence of calcium oxalate renal calculi in rat [27–30]. Liu *et al.,* demonstrated that organic selenium decreased ethylene glycol formation which impel calcium oxalate renal calculi *via* repressing the expression of osteopontin (OPN) and improving antioxidant capacity in dogs. However, another study reported that selenium supplementation did not affect the level of urinary oxalate in dogs. It seems that different animal species which is used in these studies may be the reason of these discrepancies [23].

Daudon *et al.,* reported that below cut off 3000, the stone recurrence chance is low. When the crystal volume is more than 3000, the chance of stone recurrence increases significantly. However, relation between the chance of stone recurrence and crystal volume was not evaluated in current study [13].

## 5. Conclusion

According to results of this study, selenium treatment affected cystine crystal volume. It seems that selenium had the potential value to alleviate the volume of cystine crystal. Therefore, since reducing of cystine crystal volume decreases crystal formation, selenium may be effective to cure patients with cystinuria. However, age, sex and type of medical procedures did not affect cysteine crystal volume. Furthermore, it is proposed that relation between cyctine crystal volume and stone recurrence was evaluated in future study.

## Conflict of interest statement

The authors disclose no conflicts of interest.
